# ROBITAL Atherectomy via 5-F Guide for Calcified Left Main Disease With Cardiogenic Shock

**DOI:** 10.1016/j.jaccas.2026.110355

**Published:** 2026-09-23

**Authors:** Soleiman Aria, Su Hnin Hlaing, Darren L. Walters, Rustem Dautov

**Affiliations:** a Heart and Lung Institute, The Prince Charles Hospital, Chermside, Queensland, Australia; b School of Medicine, University of Queensland, Brisbane, Queensland, Australia

**Keywords:** orbital atherectomy, robital atherectomy, rotational atherectomy

## Abstract

**Background:**

Sequential ROtational and orBITAL (ROBITAL) atherectomy combines sequential rotational and orbital atherectomy to manage complex coronary calcium. We report its feasibility using a 5-F guiding catheter in a patient with critical left main (LM) disease when larger guides could not achieve stable engagement.

**Case Summary:**

An 83-year-old woman presented with cardiogenic shock, pulmonary edema, and severe calcified ostial/distal LM disease. After intra-aortic balloon pump insertion, only a 5-F JL 3.5 guide achieved coaxial engagement. ROBITAL atherectomy allowed adequate calcium modification, allowing intravascular ultrasound-guided percutaneous coronary intervention. She recovered fully and remained well 3 years later.

**Discussion:**

Limited literature supports 5-F calcium modification strategies. This case demonstrates ROBITAL atherectomy via a 5-F guide for unstable LM disease when larger guides failed to engage the LM.

**Take-Home Message:**

ROBITAL atherectomy enables complex calcium modification through a 5-F guiding catheter, extending options for patients with only limited radial access and when larger guides cannot engage the coronaries.

## Visual Summary

**Figure:**
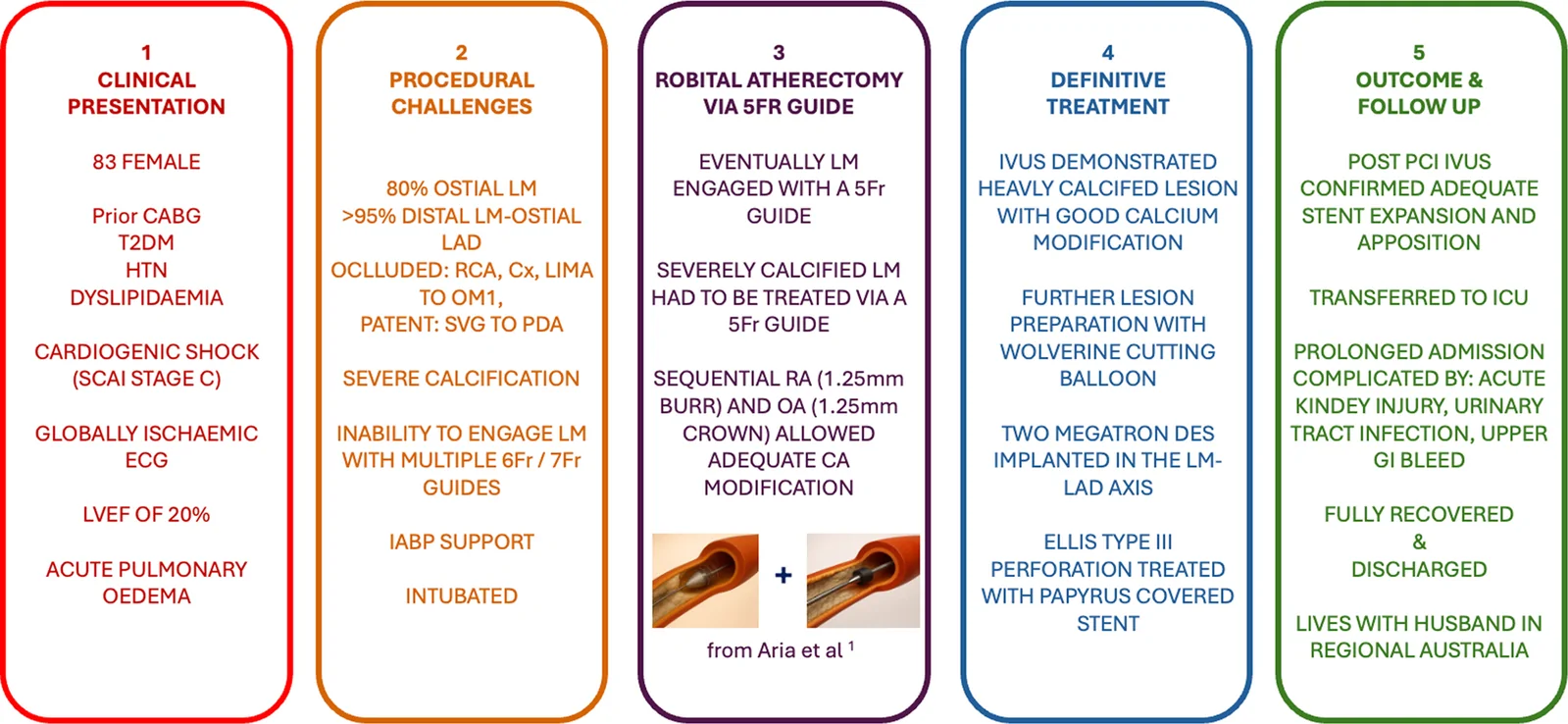

ROBITAL atherectomy is a recently described technique for managing complex, heavily calcified coronary lesions in challenging anatomies including angulated ostial left circumflex (LCx), left main (LM) bifurcations with nodular calcium, long right coronary artery (RCA) segments and cases of in-stent restenosis with underexpansion.^1^ The strategy combines the complementary mechanisms of rotational atherectomy (RA) and orbital atherectomy (OA). When OA is preferred, it is desirable to undertake it in a retrograde fashion to improve atherectomy and reduce complications. However, in heavily calcified lesions, severe stenosis may prevent the initial OA crown delivery. In such cases, a small RA burr can first create a channel through forward plaque modification, facilitating OA crown passage. OA can then be performed, allowing bidirectional sanding of calcium against the vessel wall laterally, with effective diameter increasing according to the operating speed.^1^

In our original ROBITAL series, patients had distal left main Medina 1,1,1 bifurcation disease, with clinical presentations ranging from stable angina to unstable angina and non-ST-segment elevation myocardial infarction. All remained hemodynamically stable before, during, and after percutaneous coronary intervention (PCI). Procedures were performed using 7-F or 8-F guiding catheters to ensure adequate support and device compatibility. Although OA uses a single 1.25 mm crown and is typically compatible with a 6-F guiding catheter per the manufacturer's recommendation,^2^ RA burr sizes range from 1.25 to 2.5 mm, with larger burrs often necessitating 7-F or 8-F guiding catheters, whereas smaller burrs can be used through 6-F guides.^1^

In this case report, we demonstrate the feasibility of the ROBITAL technique via a 5-F guiding catheter in a patient with severely calcified LM disease complicated by cardiogenic shock.

## Case Presentation

### History of presentation

An 83-year-old woman presented to a regional hospital with cardiogenic shock and a globally ischemic electrogram suggestive of LM coronary artery stenosis.

### Past medical history

Her medical history was significant for prior coronary artery bypass graft with left internal mammary artery (LIMA) to obtuse marginal (OM1) and saphenous vein graft to posterior descending artery, preserved left ventricular ejection fraction of 56%, hypertension, dyslipidemia, and type 2 diabetes mellitus.

A coronary angiogram performed 5 years earlier, 2 years after the coronary artery bypass graft, demonstrated 20% LM stenosis, mild diffuse left anterior descending (LAD) disease, and an 80% long, calcified mid Cx lesion that was difficult to wire. The OM1 and RCA were occluded but previously grafted. The LIMA-to-OM1 graft had poor TIMI flow grade 2 with 2 tandem lesions in the LIMA graft itself, while the saphenous vein graft to the posterior descending artery was patent. Representative still angiographic images are presented in Figures 1A to 1F, with the corresponding dynamic angiographic sequences provided in Videos 1A to 1F.

**Figure 1:**
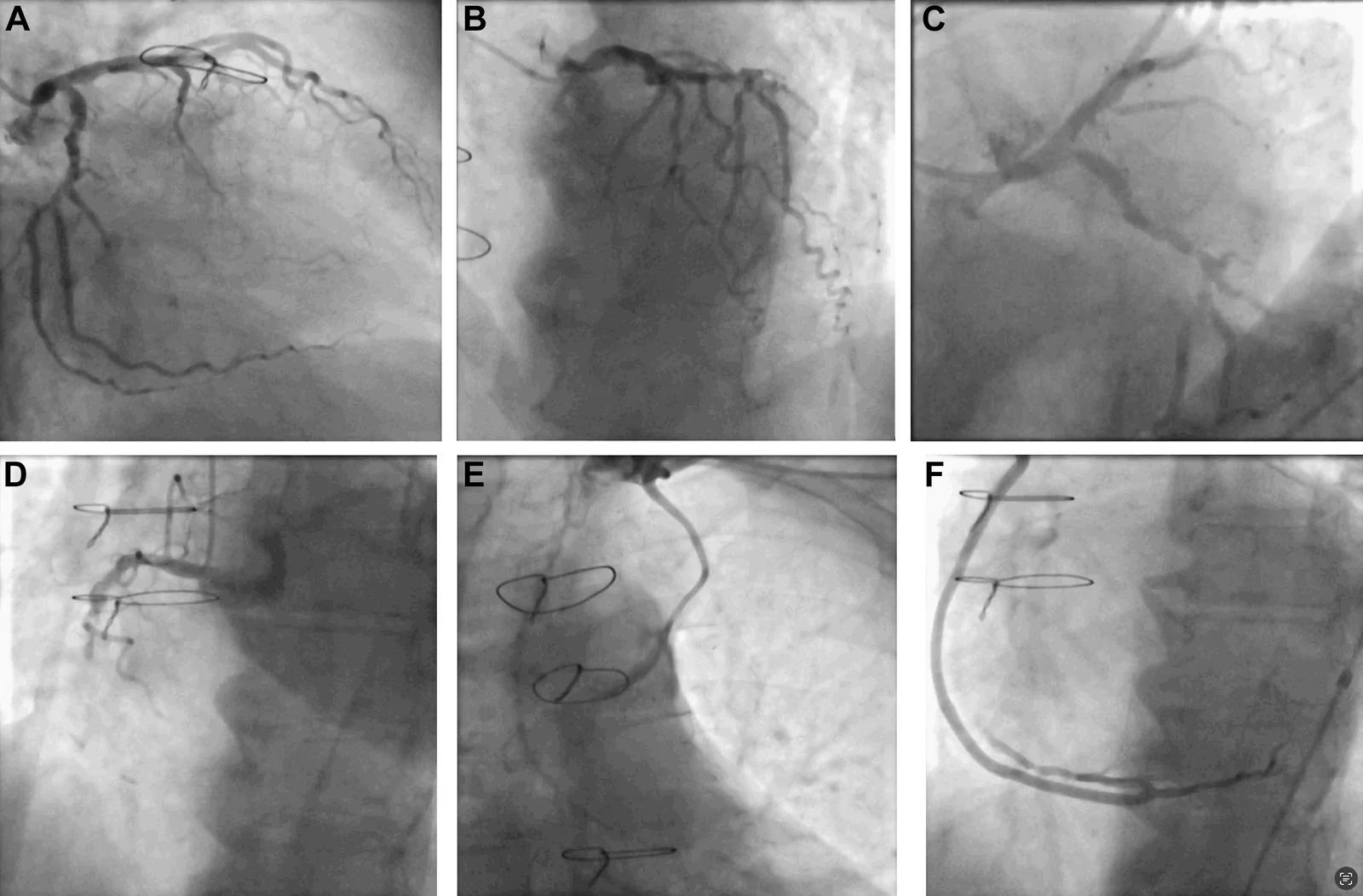
Previous Coronary Angiogram Coronary angiogram from 5 years earlier. (A to D) Native coronary artery disease. (E) Left internal mammary artery to obtuse marginal graft with tandem lesions. (F) Patent saphenous vein graft to posterior descending artery graft.

During the index admission, after thrombolysis in the regional hospital, the patient was transferred to our center via helicopter. On arrival, she was agitated and dyspneic with acute pulmonary edema.

### Investigations

The electrocardiogram remained unchanged and is shown in Figure 2D. A bedside echocardiogram showed a left ventricular ejection fraction of 20% and moderate mitral regurgitation. The patient was emergently intubated in the catheterization laboratory before angioplasty.

**Figure 2:**
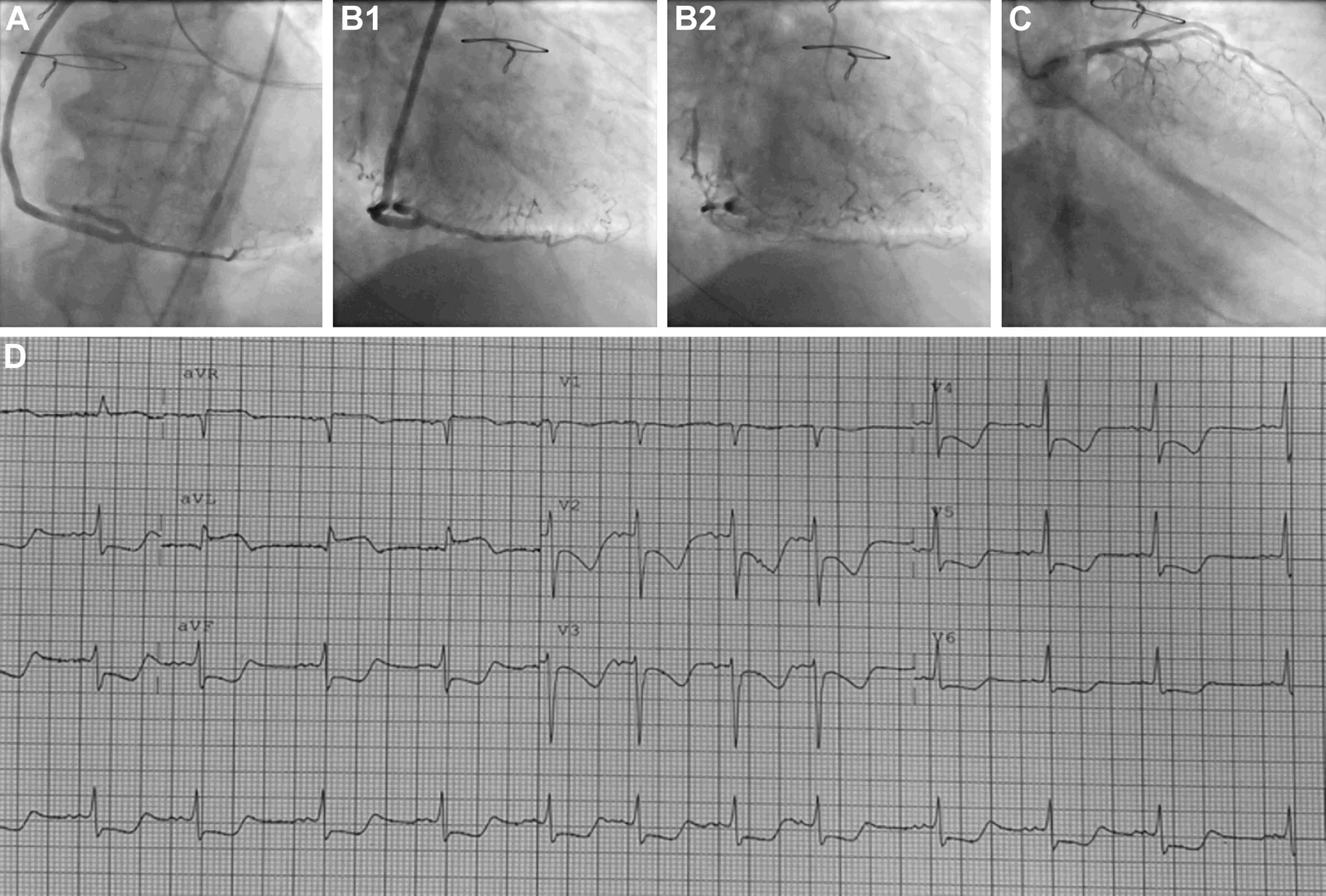
Index Admission Coronary Angiogram (A and B) Saphenous vein graft (SVG) to posterior descending artery graft (PDA), (B) SVG to PDA's collaterals to the left system. (C) Severely calcified left main disease. (D) Electrocardiogram.

### Management

Right femoral arterial access was obtained with a 9-F sheath, and a 40 cc intra-aortic balloon pump was inserted for upfront hemodynamic support. Left femoral arterial access was also obtained using a 7-F sheath. Diagnostic angiography demonstrated an 80% ostial LM stenosis with a >95% lesion in distal LM-ostial LAD. The LCx was completely occluded at the ostium, consistent with a chronic total occlusion. The LIMA to OM1 was not engaged as this was known to be occluded. The RCA was a chronic total occlusion with a patent venous graft. The latter also supplied collateral channels to the LCx. Representative still angiographic images are presented in Figures 2A to 2C, with the corresponding dynamic angiographic sequences provided in Videos 2A to 2C.

Engagement of the LM was challenging via femoral approach using multiple 7-F and 6-F guiding catheters. Eventually, the LM was successfully engaged using a 5-F JL 3.5 Zuma guiding catheter (Medtronic) via a femoral approach. LM-LAD axis was wired with Whisper wire (Abbott Vascular) into the first septal with 135 mm Turnpike LP (Teleflex) support. RA was performed using a 1.25 mm burr over an Extra Support rotawire (Boston Scientific) at 165,000 rpm, with 10 passes and a 244-second dwell time to create an initial channel. The Extra Support rotawire was then exchanged for a Viper wire (Abbott Vascular) via Turnpike LP. OA was performed with a 1.25 mm crown at 80,000 and 120,000 rpm with 11 passes and 291 seconds of dwell time. Representative cine angiography of the RA and OA is shown in Videos 3A and 3B. Viper wire was exchanged for BHW wire (Abbott Vascular), and a 5-F OptiCross 40 MHz intravascular ultrasound (IVUS) (Boston Scientific) demonstrated a heavily calcified LM disease with good calcium modification. Representative IVUS images are shown in Figure 3 with corresponding IVUS run in Video 4A. Further lesion preparation was performed with a 3.5 x 10 mm Wolverine cutting balloon (Boston Scientific) at 20 atm. A 4.5 x 32 mm Megatron drug-eluting stent (DES) failed to cross the lesion, which is shown in Video 3C. Further preparation with a 3.5 x 15 mm non-compliant (NC) balloon allowed successful implantation of a 4.0 x 20 mm Megatron DES followed by a second 4.5 x 12 mm Megatron DES, in the LM. An Ellis type III coronary perforation^3^ occurred and was sealed with a 4.0 x 20 mm Papyrus covered stent. These final stages are demonstrated in Video 5. Postintervention IVUS (Video 4B) confirmed adequate stent expansion and apposition throughout the treated segment.

**Figure 3:**
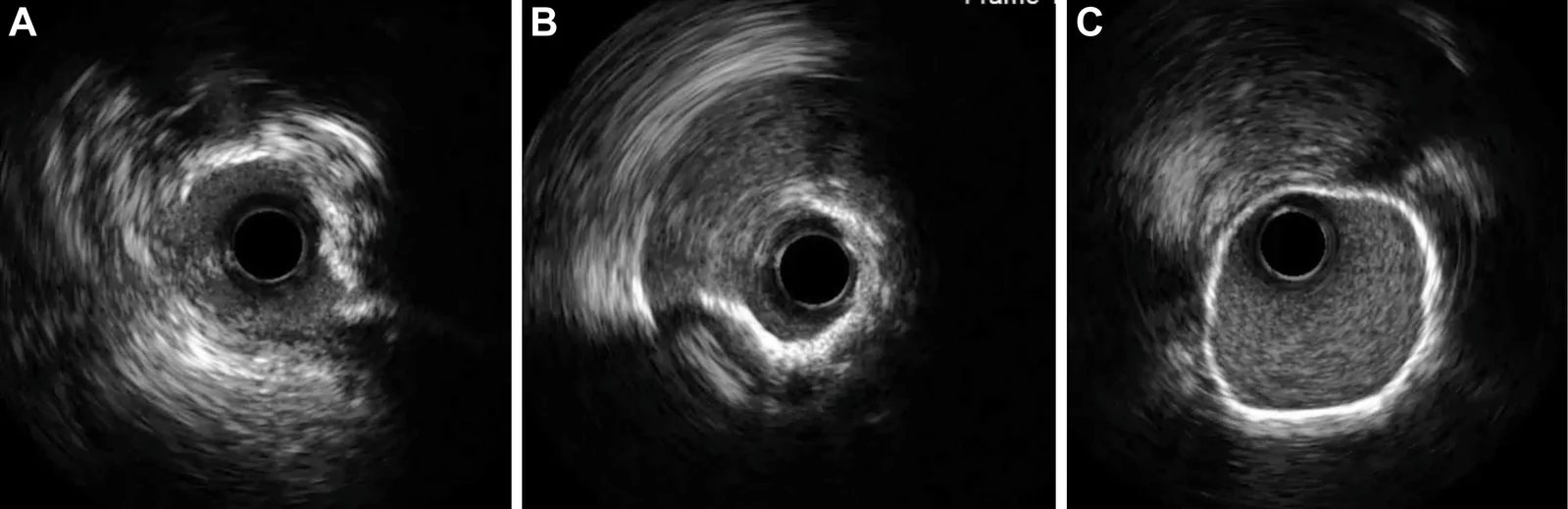
Intravascular Imaging (A and B) Intravascular ultrasound images of heavily calcified left main disease post-Ca modification. (C) Circumferential superficial calcification in left anterior descending.

### Outcome and follow-up

The patient was transferred to the intensive care unit. She had a prolonged admission complicated by acute kidney injury, urinary tract infection, delirium, and upper gastrointestinal bleed. Eventually she made a full recovery and was discharged. Three years later, she remains well and lives with her husband in coastal regional Australia.

## Discussion

This case demonstrates the feasibility of the ROBITAL technique in an acutely unstable patient with cardiogenic shock secondary to severely calcified ostial LM disease. It highlights that rapid and effective calcium modification can be achieved in an emergent setting using a 5-F catheter system. By combining the complementary mechanisms of RA and OA, the technique allows progressive plaque modification and facilitates treatment of wire-crossable but device-uncrossable lesions with extensive nodular calcium.

A notable procedural challenge in this case was engagement of the heavily calcified ostial LM. Wiring of the LM from a distance led to the guide catheter disengagement as the workhorse wire was hitting the severe distal LM lesion. Multiple attempts using standard 7-F and 6-F guiding catheters despite a femoral approach were unsuccessful. Ultimately, a 5-F JL 3.5 guide via femoral approach achieved stable engagement. Although 5-F guides offer less support and device compatibility, their reduced external diameter may aid engagement and improved coaxial alignment in tight ostial lesions.^4^ A 5-F guide can be exchanged for a larger guide over a coronary wire using an extension or in combination with a 0.035 260 cm wire. However, in patients with small radial arteries or severe femoral or iliac artery calcification, upsizing may not be feasible, leaving the operator to manage complex calcified lesions with a 5-F system.

The literature on 5-F guiding catheters in combination with calcium-modification devices is limited. Allouch et al^5^ reported the feasibility of transradial RA using a 5-F guiding catheter with a 1.25 mm burr. More recently, Kawamura et al^2^ evaluated OA through a 5-F guiding catheter in patients with heavily calcified coronary lesions. OA via a 5-F guide achieved 100% procedural success with acceptable complication rates. Similarly, Sherif et al^6^ demonstrated the feasibility and safety of transradial excimer laser atherectomy for chronic total occlusion using a 5-F sheath system, further supporting the potential of low-profile transradial calcium modification strategies. As intravascular lithotripsy can be delivered once an initial channel has been created by RA, sequential rotational atherectomy and intravascular lithotripsy (ROTASHOCK) may also represent a feasible treatment strategy.^7^ However, sequential ROtational and orBITAL atherectomy (ROBITAL) may be favored because of its greater cost-effectiveness, with an estimated cost of 5.7K AUD compared with approximately 8K AUD for ROTASHOCK in the Australian market, although costs may vary across regions globally.^1^

## Conclusions

This case demonstrates the feasibility of performing complex coronary calcium modification and PCI through a 5-F guiding catheter using the ROBITAL technique with sequential RA and OA. In this critically ill patient with severely calcified tight left main disease, IVUS-guided PCI incorporating a 1.25 mm RA burr, 1.25 mm OA crown, 3.5 mm Wolverine cutting balloon, 4.5 mm Megatron stent and 4.0 mm papyrus covered stent was successfully performed via a 5-F guiding catheter, while engaging the coronary artery with larger guiding catheters was challenging.

The applicability of this strategy, in addition to the situations where engagement with larger guiding catheters is challenging, extends to patients with small radial arteries, commonly encountered in women and Asian patients, particularly when femoral access should be avoided because of increased bleeding risk, anticoagulation therapy, severe peripheral vascular disease, or obesity.

**Take-Home Message**

• ROBITAL atherectomy enables complex calcium modification through a 5-F guiding catheter, extending options for patients with only limited radial access and those when larger guides cannot engage the coronaries.

**Visual Summary:**
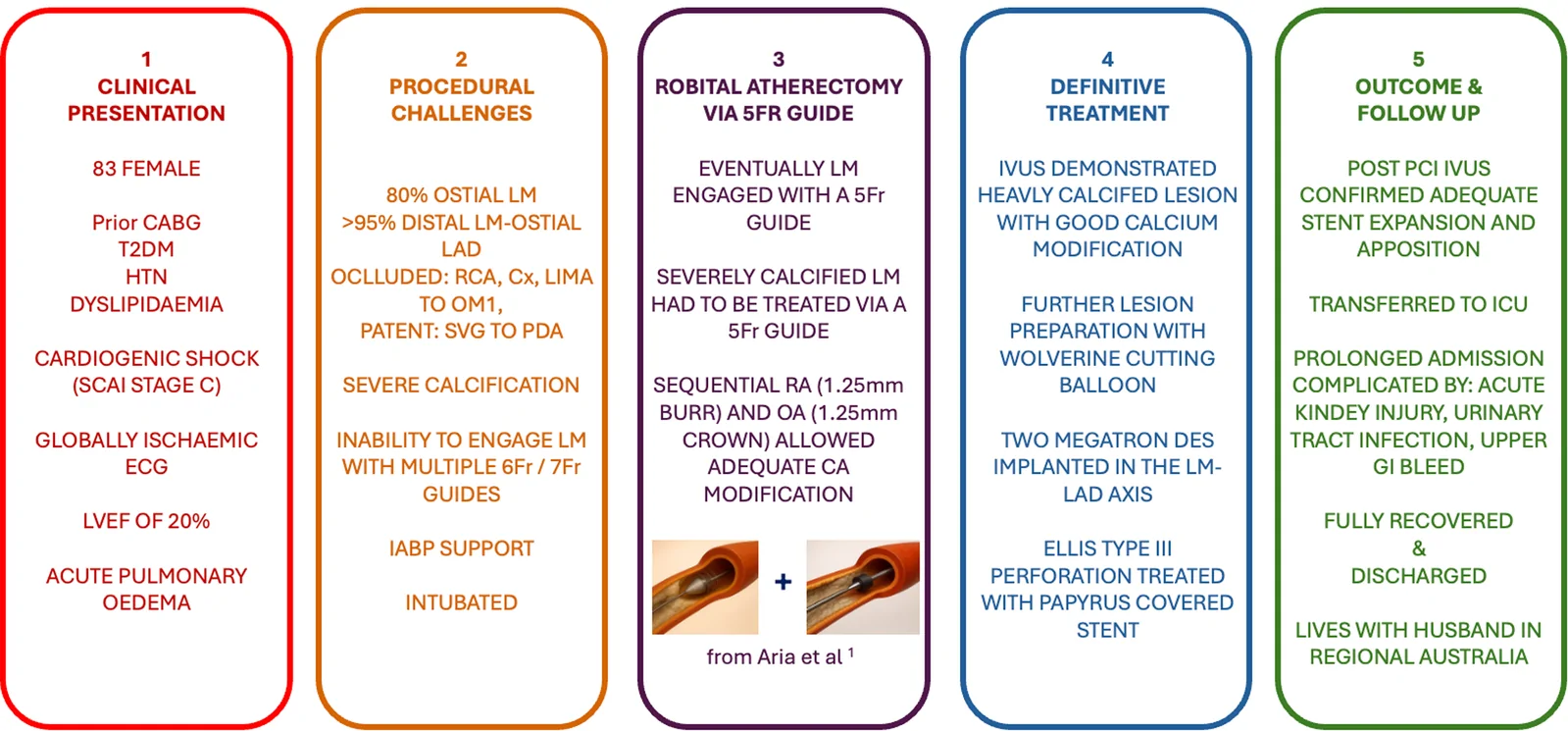
In a Patient With Cardiogenic Shock Requiring Urgent Revascularization, Conventional 6-F and 7-F Guide Catheters Failed to Engage the Left Main Coronary Artery Only a 5-F Guide Achieved Engagement A 1.25 mm Rotational atherectomy (RA) burr created an initial channel, enabling orbital atherectomy (OA) crown delivery. Sequential RA and OA, ROBITAL atherectomy, facilitated successful percutaneous coronary intervention (PCI) of a severely calcified left main lesion. CABG = coronary artery bypass graft; Cx = circumflex; ECG = electrocardiogram; GI = gastrointestinal; HTN = hypertension; IABP = intra-aortic balloon pump; ICU = intensive care unit; IVUS = intravascular ultrasound; LAD = left anterior descending; LIMA = left internal mammary artery; LM = left main; LVEF = left ventricular ejection fraction; OM1 = obtuse marginal; PDA = posterior descending artery; RCA = right coronary artery; SVG = saphenous vein graft; T2DM = tyep 2 diabetes mellitus.

## Funding Support and Author Disclosures

Dr Walters has received proctorship honoraria and has consulted for Abbott. Dr Dautov has received speaker and proctorship honoraria from Abbott Vascular and Boston Scientific. All other authors have reported that they have no relationships relevant to the contents of this paper to disclose.

## Appendix

Video 1 Previous Coronary Angiogram Corresponding dynamic angiographic sequences to Figure 1, A to D: native coronary artery disease, E: left internal mammary artery-obtuse marginal (LIMA-OM1) graft with tandem lesions, F: Patent saphenous vein graft-posterior descending artery (SVG-PDA) graft.

Video 2 Pre-Intervention Coronary Angiogram With IABP in In Situ Corresponding dynamic angiographic sequences to Figure 2, A and B: saphenous vein graft (SVG) to posterior descending artery (PDA) and its collaterals to the left system, C: Severely calcified left main (LM) disease.

Video 3 ROBITAL Atherectomy A: Rotational atherectomy (RA), B: orbital atherectomy (OA), C: Failure of first drug-eluting stent (DES) delivery.

Video 4 Pre and Post PCI IVUS A: Extensive superficial calcium with areas of calcium fracture and plaque modification. B: Final intravascular ultrasound (IVUS) run demonstrating adequate stent expansion and apposition throughout the treated segment.

Video 5 PCI, Perforation and Papyrus Deployment A: Deployment of first successful drug-eluting stent (DES), B: deployment of second DES, C: perforation, D: papyrus stent Deployment
